# Water Diffusivity Changes Along the Perivascular Space After Lumboperitoneal Shunt Surgery in Idiopathic Normal Pressure Hydrocephalus

**DOI:** 10.3389/fneur.2022.843883

**Published:** 2022-02-28

**Authors:** Junko Kikuta, Koji Kamagata, Toshiaki Taoka, Kaito Takabayashi, Wataru Uchida, Yuya Saito, Christina Andica, Akihiko Wada, Kaito Kawamura, Chihiro Akiba, Madoka Nakajima, Masakazu Miyajima, Shinji Naganawa, Shigeki Aoki

**Affiliations:** ^1^Department of Radiology, Juntendo University Graduate School of Medicine, Bunkyo-ku, Japan; ^2^Department of Innovative Biomedical Visualization, Graduate School of Medicine, Nagoya University, Nagoya, Japan; ^3^Department of Neurosurgery, Juntendo University Faculty of Medicine, Bunkyo-ku, Japan; ^4^Department of Neurosurgery, Juntendo Tokyo Koto Geriatric Medical Center, Koto-ku, Japan; ^5^Department of Radiology, Nagoya University Graduate School of Medicine, Nagoya, Japan

**Keywords:** idiopathic normal pressure hydrocephalus, ALPS index, lumboperitoneal shunt surgery, glymphatic system, diffusion-weighted imaging, cerebrospinal fluid, interstitial fluid

## Abstract

**Background:**

The aim of this study was to evaluate the water diffusivity changes along the perivascular space after lumboperitoneal shunt (LPS) surgery in idiopathic normal pressure hydrocephalus.

**Methods:**

Nine patients diagnosed with idiopathic normal pressure hydrocephalus (iNPH; three men and six women, mean age ± SD = 75.22 ± 5.12 years) according to the guidelines for iNPH in Japan were included in the study. Post-LPS surgery, six patients with iNPH who exhibited improvement in symptoms were defined as responder subjects, while three patients with iNPH who did not were defined as non-responder subjects. We calculated the mean analysis along the perivascular space (ALPS) index of the left and right hemispheres and compared the differences between pre- and post-LPS surgery mean ALPS indices in iNPH patients. In the responder or non-responder subjects, the mean ALPS indices in the pre- and post-operative iNPH groups were compared using Wilcoxon signed-rank tests. Next, correlation analyses between pre- and post-operation changes in the mean ALPS index and clinical characteristics were conducted.

**Results:**

The mean ALPS index of the post-operative iNPH group was significantly higher than that of the pre-operative iNPH group (*p* = 0.021). In responder subjects, the mean ALPS index of the post-operative iNPH group was significantly higher than that of the pre-operative iNPH group (*p* = 0.046). On the other hand, in the non-responder subjects, the mean ALPS index of the post-operative iNPH group was not significantly different compared to the pre-operative iNPH group (*p* = 0.285). The mean ALPS index change was not significantly correlated with changes in the Mini-Mental State Examination (MMSE) score (*r* = −0.218, *p* = 0.574), Frontal Assessment Battery (FAB) score (*r* = 0.185, *p* = 0.634), Trail Making Test A (TMTA) score (*r* = 0.250, *p* = 0.516), and Evans' index (*r* = 0.109, *p* = 0.780). In responder subjects, the mean ALPS index change was significantly correlated with Evans' index in pre-operative patients with iNPH (*r* = 0.841, *p* = 0.036).

**Conclusion:**

This study demonstrates the improved water diffusivity along perivascular space in patients with iNPH after LPS surgery. This could be indicative of glymphatic function recovery following LPS surgery.

## Introduction

Idiopathic normal pressure hydrocephalus (iNPH) is accompanied by gait dysfunction, cognitive impairment, and urinary incontinence in the elderly (1). The prevalence of iNPH varies between populations and can be as high as between 1.3 and 2.1% for a population aged between 65 and 79, and 8.9% for those above 80 years of age (2, 3). iNPH is characterized by dilation of the cerebral ventricles, normal cerebrospinal fluid (CSF) pressure, and mostly symptomatic improvement after CSF diversion procedures (4). In a prospective study of iNPH, shunt surgery efficacy was reported to be approximately 80% in cases diagnosed with one or more of these symptoms (5). Besides, lumboperitoneal shunt (LPS) surgery is a minimally invasive approach and a popular treatment option for patients with iNPH in Japan (6, 7). Nagajima et al. (7) reported that the treatment efficacy of LPS against iNPH was not different from what is obtained following ventriculoperitoneal shunting. However, without treatment, the disease progresses, symptoms worsen and the probability of favorable outcomes from shunt surgery diminishes (8).

Characteristic features of iNPH that can be identified in imaging include dilatation of the Sylvian fissure, narrowing of the CSF space in the parietal region, and callosal angle. The effectiveness of shunt placement for the treatment of iNPH has been evaluated using CT or MRI. Kitagaki et al. (9) showed that in five patients with iNPH, the mean post-operative CSF volumes were significantly decreased in the Sylvian space and in the ventricle, marginally decreased in the basal cistern, and significantly increased in the suprasylvian space as compared with the pre-operative volumes. Anderson et al. (10) reported that the ventricular volumes of 10 (91%) out of 11 patients with iNPH who underwent shunt surgery were decreased, with a mean change rate of 39%. Hiraoka et al. (11) found that the decrease in ventricular volumes had a significant correlation to clinical improvement after shunt surgery.

In iNPH, the accumulation of amyloid-β is known to be one of the causes of impairment in cognitive symptoms. The accumulation of interstitial amyloid-β is a characteristic feature of both iNPH and Alzheimer's disease (AD). Typically, compared to patients with AD who have increased production of amyloid-β, patients with iNPH are believed to have impaired CSF absorption and excretion, thereby resulting in the accumulation of amyloid-β in the brain (9). However, there is also iNPH with AD pathology, the symptom is more complicated. The accumulation of interstitial amyloid-β is a pathological feature that correlates with poor shunt responsiveness in patients with iNPH (10). Therefore, studies on clearance pathways of waste products in iNPH are very important for the development of more effective treatments for these patients. There exist some gaps in our understanding of the mechanisms of the clearance system by CSF dynamics in iNPH.

Recently the glymphatic system hypothesis has been put forward to help to evaluate the elimination of waste solutes, such as amyloid-β and tau protein, in the brain parenchyma (12). The glymphatic system is essential for tissue homeostasis and is mediated by the CSF-interstitial fluid (ISF) exchange pathway (13). The exchange of fluids and their solutes between the CSF and ISF is dominated by convection and diffusion. Convection is characterized by solutes following the movement of their solvent (bulk flow). Diffusion is driven mainly by Brownian motion and characterized by solute movement being symmetric from a higher to a lower concentration. Aquaporin-4 water channels on astrocytic end-feet have been proposed to support perivascular fluid and solute movement along the glymphatic system (12). The glymphatic system is believed to be involved in AD, Parkinson's disease, diabetes, Meniere's disease, traumatic cerebral damage, iNPH, and various other diseases (13). Iliff et al. (14) demonstrated in mice using labeled CSF *via* injection of a fluorescent tracer into the cisterna magna that the CSF enters the brain along the cortical pial arteries. Several studies have assessed CSF dynamics primarily using tracers through intrathecal or intravenous injection of a gadolinium-based contrast agent (GBCA) and observations using MRI (15–19). As a part of MRI tracer studies using stable isotopes to evaluate CSF motion, the analysis of H_2_^17^O has also been reported. Kudo et al. (20) utilized dynamic steady-state sequences to detect the T2-shortening effect by H_2_^17^O.

Taoka et al. (21) introduced the analysis along the perivascular space (ALPS) method, which is calculated using diffusion-tensor imaging as a non-invasive tool to evaluate the glymphatic system of living humans. This method assumes that diffusion may have an essential role in fluid transport in the brain parenchyma. Their study showed that in AD, the ALPS index was positively correlated with the Mini-Mental State Examination (MMSE) score (21). ALPS method was also reported to be robust under fixed imaging method and parameters even when different scanners were used (22). Subsequently, there have been reports of the use of the ALPS method for the assessment of different conditions in living patients, such as AD (21), iNPH (23, 24), diabetes (25), hypertension (26), Parkinson's disease (27–29), stroke (30), multiple sclerosis (31), and tumor-associated brain edema (32, 33). Interestingly, recently Zhang et al. (34) using intrathecal administration of gadolinium in patients with small vessel disease (*r* = −0.772 to −0.844, *p* < 0.001) demonstrated that the ALPS index is significantly associated with glymphatic clearance. This study strongly supports the clinical use of the ALPS index as a glymphatic biomarker. Furthermore, to our knowledge, there are only two studies that have explored the glymphatic function in patients with iNPH, using the ALPS method (22, 23). Yokota et al. (22) showed that ALPS indices were lower in both pseudo-iNPH and iNPH patients than in healthy controls. Bae et al. (23) also reported the ALPS index to be significantly lower in iNPH patients compared to controls (*p* < 0.0001). The ALPS index was also significantly lower in the iNPH group, which did not show a treatment response that could be detected by diagnostic CSF drainage (lumbar puncture of 50 cc) as opposed to the group, which showed symptomatic improvement (*p* < 0.0001). These reports indicate that patients with iNPH exhibit lower water diffusivity in the direction of the perivascular space in the living human brain than healthy subjects. Bae's study examined patients with iNPH who only underwent the diagnostic CSF drainage (23); however, changes of diffusivity in the direction of the perivascular space post-shunt surgery have not been evaluated yet. Hence, this study aimed to evaluate post-LPS surgery changes in the ALPS index in patients with iNPH.

## Methods

### Study Participants

Nine patients diagnosed who were with iNPH (three men and six women, mean age ± SD = 75.22 ± 5.12 years) according to the guidelines for iNPH in Japan were included in this study (35). The following inclusion criteria were adopted: (1) patients aged between 60 and 85 years; (2) the presence of the triad of symptoms, which were measurable on the iNPH grading scale (35); (3) both ventriculomegaly with an Evans' index >0.3 and high-convexity and medial subarachnoid space tightness on coronal MR images; and (4) the absence of disorders known to produce ventriculomegaly. The exclusion criteria were as follows: (1) complications with locomotor disorders (bone and joint disorders, etc.), visceral disorders (heart failure, etc.), and other psychiatric disorders; (2) complications with a hemorrhagic predisposition, abnormal blood coagulation, or hemorrhagic disease (cerebral hemorrhage, subarachnoid hemorrhage, and active peptic ulcer); (3) patients with hepatic dysfunction that may affect the clinical symptoms of iNPH, such as hepatic coma (aspartate transaminase [AST] or alanine aminotransferase [ALT] 100 U/L or more within 3 months of consent date); and (4) patients with renal dysfunction that required artificial dialysis (serum creatinine ≧2.0 mg/dl within 3 months of consent date). Evans' index, MMSE, Frontal Assessment Battery (FAB), and Trail Making Test A (TMTA) were conducted in patients with iNPH for pre- and post-LPS surgery. The clinical and MRI data of patients' post-LPS surgery were obtained within 1 year of surgery. Table 1 shows the demographic characteristics of the subjects.

**Table 1:** Demographic characteristics of subjects.

	**Pre-operation iNPH group**	**Post-operation iNPH group**	**Pre-operation vs. Post-operation *P*-values**
Sex (Men / Female)	3/6		
Age	75.22 ± 5.12		
Evans' index	0.33 ± 0.03	0.31 ± 0.03	0.012
MMSE	24.11 ± 3.48	25.67 ± 2.40	0.228
FAB	12.78 ± 3.67	14.00 ± 3.12	0.392
TMTA	104.78 ± 31.74	91.11 ± 34.93	0.351
Fazekas scale			
PVH	2.33 ± 0.50	2.33 ± 0.50	1
DSWMH	2.33 ± 0.50	2.33 ± 0.50	1

*MMSE, Mini-Mental Statement Examination; FAB, Frontal Assessment Battery; TMTA, Trail Making Test A; PVH, Periventricular hyperintensity; DSWMH, Deep and subcortical white matter hyperintensity*.

### MRI Acquisition

All MRI data were acquired using a 3T MRI scanner (Achieva Quasar Dual; Philips Medical Systems, Best, The Netherlands). We performed fluid-attenuated inversion recovery (FLAIR) imaging. A magnetization-prepared rapid acquisition gradient-echo (MPRAGE) sequence was collected as a high resolution, three-dimensional structural image. Diffusion-weighted imaging (DWI) with 32 non-collinear directions was acquired using a single-shot spin-echo echo-planar imaging sequence. Echo-planar images were acquired using a b value of 1,000 s/mm^2^ along 32 isotropic diffusion gradients in the anterior-posterior phase-encoding direction. Each DWI acquisition was completed with a b = 0 image. We also acquired standard and reverse phase-encoded blipped images with no diffusion weighting (blip-up and blip-down) to correct for magnetic susceptibility-induced distortions related to the echo-planar image acquisitions. Within ~1 year of shunting, the second MRI scan was performed on each participant. The acquisition parameters of FLAIR, MPRAGE, and DWI are presented in Table 2. Periventricular hyperintensity and deep white matter hyperintensity evaluations using the Fazekas scale (36) and Evans' index based on axial FLAIR imaging were conducted by an experienced neuroradiologist (JK).

**Table 2:** Acquisition parameters for MPRAGE, DWI, and FLAIR.

**Imaging sequence**	**MPRAGE**	**DWI**	**FLAIR**
TR (ms)	15	3,000	10,000
TE (ms)	3.52	80	120
Slice thickness (mm)	0.86	5	5
Voxel size (mm^3^)	0.8125 × 0.8125 × 0.86	2 × 2 × 5	0.72 × 1.13 × 5
FOV (mm)	260 × 260	256 × 256	230 × 200
b-values (s/mm^2^)		0, 1,000	
Acquisition time (min:s)	5:14	2:43	2:40

*DWI, Diffusion-weighted image; FOV, Field of view; MPRAGE, Magnetization-prepared rapid acquisition gradient-echo; TR, Repetition time; TE, Echo time*.

### DWI Processing

Diffusion-weighted imaging data were processed using FMRIB Software Library (FSL; Oxford Center for Functional MRI of the Brain, Oxford, UK; www.fmrib.ox.ac.uk/fsl;) version 6.0 (37). The DWI data were corrected for susceptibility-induced geometric distortions, eddy current distortions, and inter-volume subject motion using EDDY and TOPUP toolboxes (38). Diffusivity maps of each subject in the direction of the x-axis (right-left; *Dxx*), y-axis (anterior-posterior; *Dyy*), and z-axis (inferior-superior; *Dzz*) were obtained. Fractional anisotropy (FA) maps of all participants were also created and aligned into the FMRIB58_FA standard space using FSL's linear and non-linear registration tools.

### Region of Interest (ROI) Placement and ALPS Index Calculation

**Figure 1:**
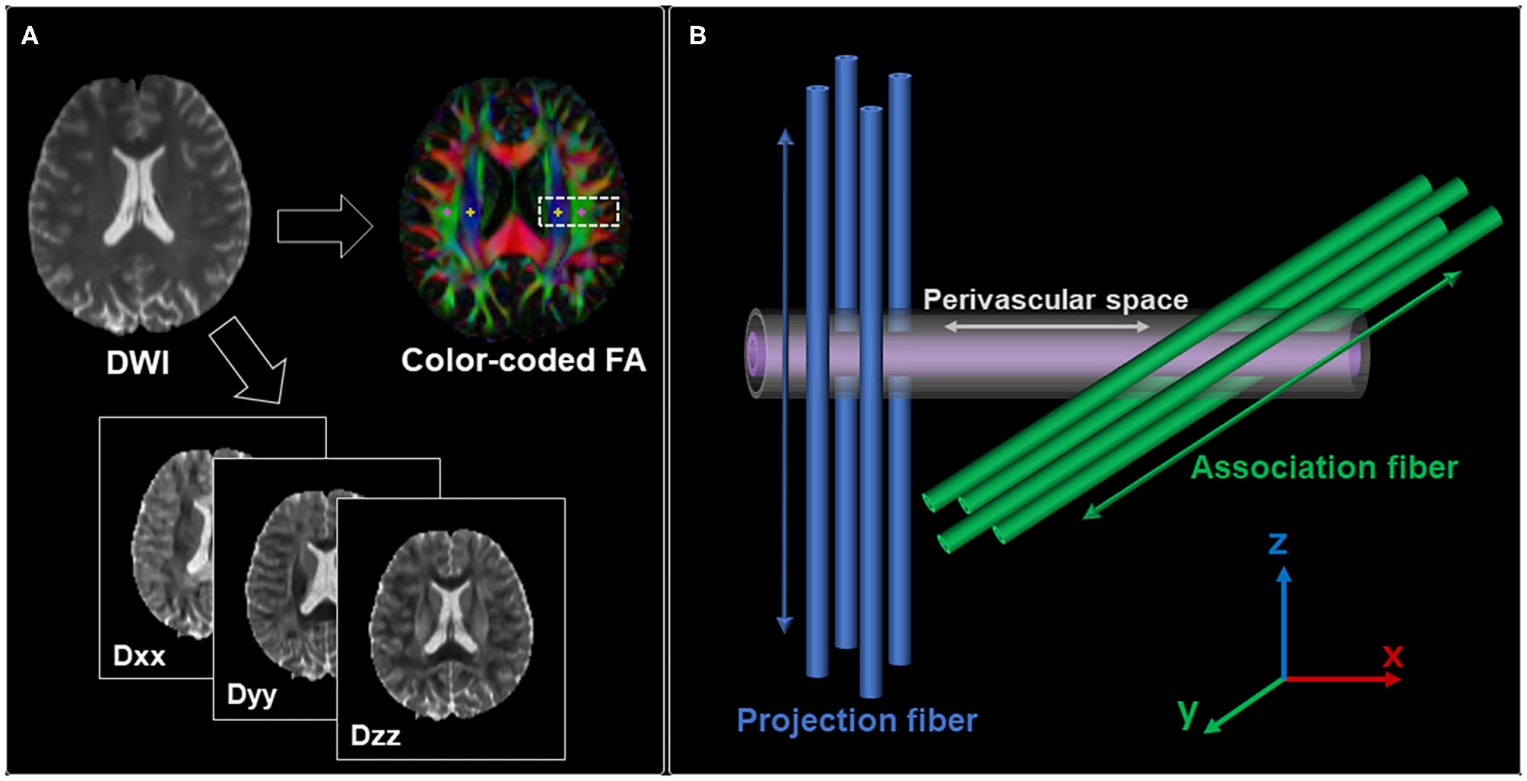
**(A)** Region of interest (ROI) placement for the calculation of the ALPS index. ROIs with a size of 5 × 5 mm^2^ were manually placed in the projection area (yellow) and association area (pink). **(B)** Schematic indicating the relationship between the direction of the perivascular space and the directions of the fibers. It shows that the direction of the perivascular space is perpendicular to both projection and association fibers.

Using each subject's color-coded FA map, 5-mm-diameter ROIs were manually placed in the projection and association areas at the level of the right and left lateral ventricles (Figure 1A). These ROIs were registered to the same FA template.

The diffusivity values in the *x*-, *y*-, and *z*-axes within ROIs were obtained from each participant (Figure 1B). The ALPS index was calculated as a ratio of the mean of the x-axis diffusivity in the projection area (*Dxxproj*) and association area (*Dxxassoc*) to the mean of the y-axis diffusivity in the projection area (*Dyyproj*) and the z-axis diffusivity in the association area (*Dzzassoc*) as follows (21): $ALPS\;index=\;\frac{\;\left(Dxxproj∔Dxxassoc\right)}{\;\left(Dyyproj∔\;Dzzassoc\right).}$

An ALPS index close to 1.0 reflects minimal diffusion along the perivascular space, whereas higher values indicate greater diffusivity. The left and right ALPS indices and the mean ALPS index of the left and right hemispheres were calculated.

### Statistical Analysis

Statistical analysis was performed using SPSS Statistics version 27.0 (IBM Corporation, Armonk, NY, USA). Age, sex, Evans' index, MMSE scores, FAB scores, and TMTA scores in the pre-operative iNPH group were compared to those in the post-operative group using Fisher's exact tests or Wilcoxon signed-rank tests. Moreover, age, sex, disease duration, Evans' index, MMSE scores, FAB scores, and TMTA scores for the responder group were compared to those for the non-responder group using Fisher's exact tests or Mann–Whitney U tests. Next, the mean ALPS indices in the pre- and post-operative iNPH groups were compared using the Wilcoxon signed-rank tests. For the responder or non-responder subjects, the mean ALPS indices in the pre- and post-operative iNPH groups were compared using the Wilcoxon signed-rank tests. Besides, both pre-operation and post-operation, the mean ALPS indices between the responder and non-responder groups were compared using Mann–Whitney U tests. A value of *p* < 0.05 was considered statistically significant (two-tailed). The effect sizes were calculated using Cohen's d to evaluate the statistical power of the relationships that were determined based on inter-group comparisons. Effect sizes of 0.2, 0.5, and 0.8 were classified as small, medium, and large, respectively (39). *Post-hoc* statistical power was calculated based on the sample and observed effect sizes. In addition, changes in the mean ALPS index, Evans' index, MMSE scores, FAB scores, and TMTA scores between the pre- and post-operative iNPH groups were calculated. Then, the association between the mean ALPS index and Evans' index changes, the mean ALPS index and MMSE score changes, the mean ALPS index and FAB score changes, and the mean ALPS index and TMTA score changes were evaluated using Spearman correlation coefficients. Moreover, for the responder subjects, the association between the mean ALPS index change and Evans' index for the pre-operative iNPH group was evaluated using Spearman correlation coefficients.

## Results

Table 1 summarizes patient characteristics and all measurements. Evans' index for the post-operative iNPH group was significantly lower than that of the pre-operative iNPH group. However, age, sex, disease duration, MMSE scores, FAB scores, and TMTA scores of the post-operative iNPH group were not significantly different from those of the pre-operative group.

Table 3 shows the demographic characteristics of the responder and non-responder groups. Mori (40) reported that among the symptoms of iNPH, the symptoms of gait disturbance are most likely to occur, and the rate of improvement by shunt surgery is also high. For this reason, six patients with iNPH (one man and five women, mean age ± SD = 75.17 ± 5.04 years) who exhibited an improvement in at least the symptom of gait dysfunction following LPS surgery were defined as responder subjects. On the other hand, three iNPH (one man and two women, mean age ± SD = 75.33 ± 7.37 years) patients who did not exhibit improvement in any symptoms following LPS surgery were defined as non-responder subjects. Age, sex, Evan's index, MMSE scores, FAB scores, and TMTA scores pre-operation in the responder group were not significantly different from those of the non-responder group.

**Table 3:** Demographic characteristics of the responder and non-responder groups.

	**Responder group**	**Non-responder group**	**Responder vs. Non-responder *P*-values**
Number	6	3	
Sex (Men / Female)	1/5	1/2	1
Age	75.17 ± 5.04	75.33 ± 7.37	1
Disease duration (year)	1.5 ± 1.64	4.3 ± 2.08	0.095
Evan index (pre-ope)	0.33 ± 0.02	0.35 ± 0.03	0.167
MMSE (pre-ope)	23.33 ± 4.08	25.67 ± 1.15	0.714
FAB (pre-ope)	12.83 ± 3.82	12.67 ± 4.16	0.905
TMTA (pre-ope)	106.83 ± 38.78	100.67 ± 15.18	1
Fazekas scale			
PVH	2.50 ± 0.55	2	0.464
DSWMH	2.50 ± 0.55	2	0.464

*MMSE, Mini-Mental Statement Examination; FAB, Frontal Assessment Battery; TMTA, Trail Making Test A; PVH, Periventricular hyperintensity; DSWMH, Deep and subcortical white matter hyperintensity*.

**Figure 2:**
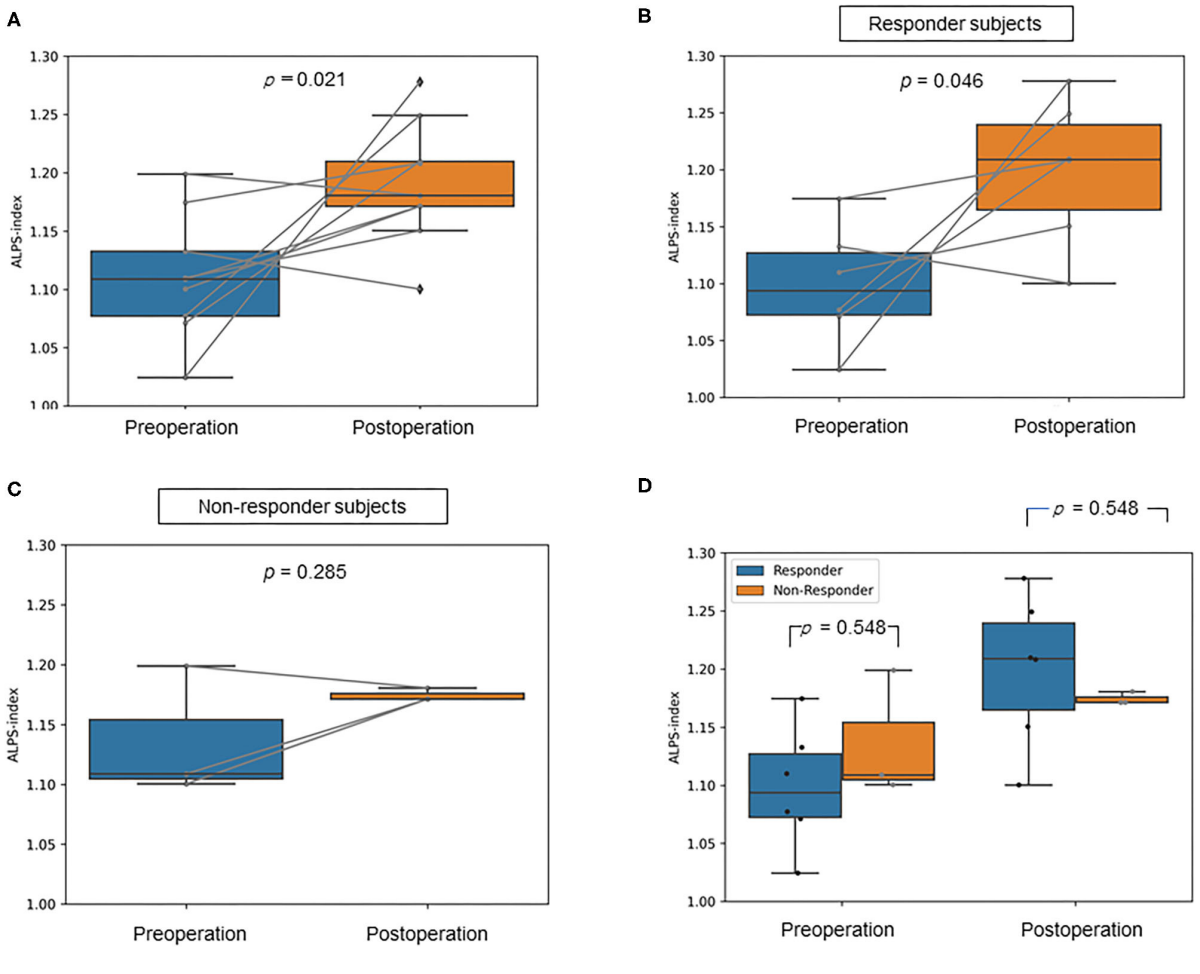
Box plots show **(A)** the difference in the mean analysis along the perivascular space (ALPS) index between the pre-operative and post-operative idiopathic normal pressure hydrocephalus (iNPH) groups, **(B)** the difference in the mean ALPS index between the pre-operative and post-operative iNPH groups in responder subjects, **(C)** the difference in the mean ALPS index between the pre-operative and post-operative iNPH groups in non-responder subjects, **(D)** the difference in the mean ALPS index between the responder and non-responder group in pre- and post-operation.

The mean ALPS index of the post-operation group was significantly higher than that of the pre-operation group (*p* = 0.021, Cohen's d = 1.51, statistical power = 0.85; Figure 2A). Additionally, for the responder subjects, the mean ALPS index of the post-operation group was significantly higher than that of the pre-operation group (*p* = 0.046, Cohen's d = 1.71, statistical power = 0.76; Figure 2B). On the other hand, in the non-responder subjects, the mean ALPS index of the post-operation group was not significantly different compared to that of the pre-operation group (*p* = 0.285, Cohen's d = 0.99, statistical power = 0.16; Figure 2C). Besides, the mean ALPS indices of the responder group were not significantly different compared to those of the non-responder group in both the pre-operation (*p* = 0.548, Cohen's d = 0.99, statistical power = 0.16) and post-operation groups (*p* = 0.548, Cohen's d = 0.99, statistical power = 0.16; Figure 2D).

**Figure 3:**
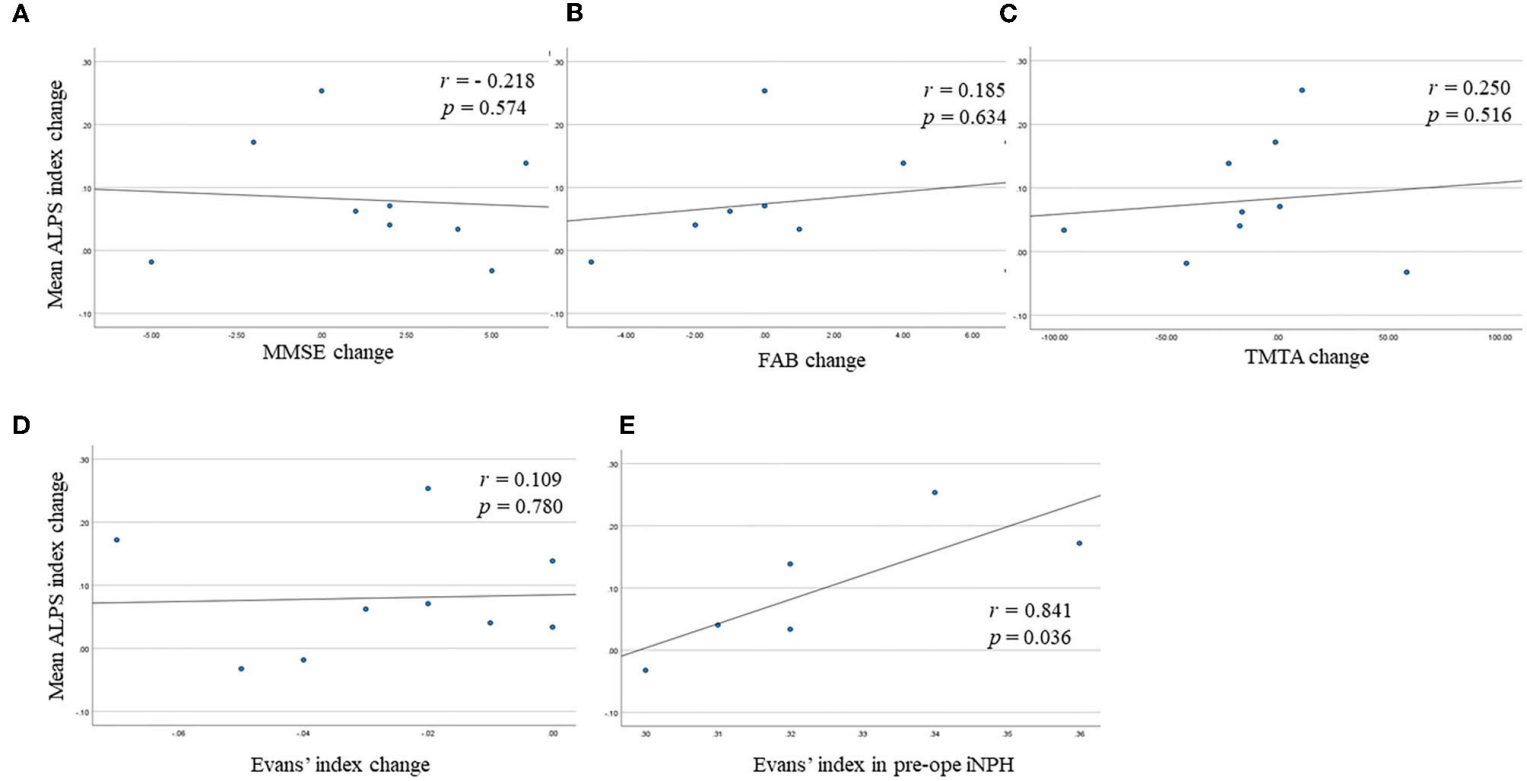
Scatter plots show **(A)** the correlation between the mean analysis along the perivascular space (ALPS) index change and Mini-Mental Statement Examination (MMSE) score change, **(B)** the correlation between the mean ALPS index change and Frontal Assessment Battery (FAB) score change, **(C)** the correlation between the mean ALPS index change and Trail Making Test A (TMTA) score change, **(D)** the correlation between the mean ALPS index change and Evans' index change, **(E)** the correlation between the mean ALPS index change and Evans' index in pre-operative idiopathic normal pressure hydrocephalus (iNPH) group in responder subjects.

Figure 3 illustrates the correlations of the mean ALPS index change with MMSE score change, FAB score change, TMTA score change, and Evans' index change. The mean ALPS index change was not significantly correlated with MMSE score change (*r* = −0.218, *p* = 0.574), FAB score change (*r* = 0.185, *p* = 0.634), TMTA score change (*r* = 0.250, *p* = 0.516), and Evans' index change (*r* = 0.109, *p* = 0.780). However, in responder subjects, the mean ALPS index change was significantly correlated with Evans' index in pre-operative iNPH patients (*r* = 0.841, *p* = 0.036).

## Discussion

This is the first reported study to have evaluated ALPS index change following LPS surgery in iNPH subjects. Our results demonstrate that the mean ALPS index of post-operative iNPH subjects was significantly higher than that of pre-operative iNPH subjects. This may be indicative of glymphatic function recovery following LPS surgery. In the responder subjects, the mean ALPS index of the post-operative iNPH group was significantly higher than that of the pre-operative iNPH group. On the other hand, in the non-responder subjects, the mean ALPS index of the post-operative iNPH group was not significantly different from that of the pre-operative group. Besides, the mean ALPS indices of the responder group were not significantly different from those of the non-responder group both pre-operation and post-operation. Notably, in the responder subjects, mean ALPS index change was significantly correlated with Evans' index in pre-operative iNPH patients. This result possibly indicates that the mean ALPS index recovered remarkably in progressive iNPH patients in the responder subjects. However, the mean ALPS index change after LPS surgery was not significantly correlated with Evans' index, MMSE score, FAB score, and TMTA score changes.

This study showed that water diffusivity in the direction of the perivascular space was improved following LPS surgery and could indicate the improvement of CSF-ISF dynamics. Several effects of shunt surgery have been previously established, such as alterations in CSF dynamics (41, 42), which can lead to secondary changes in cerebral blood flow and metabolism (43, 44) and CSF components (45, 46). Nocun et al. (47) reported that cerebral perfusion is promptly recovered after shunt surgery in about 60% of patients with iNPH. Kawamura et al. (46) have reported that CSF amyloid-β oligomers were eliminated by LPS surgery. Thus, the insertion of a shunt could dramatically alter the CSF turnover and hydrodynamic properties of the cranio-spinal system. Assessment of the role of CSF dynamics is particularly important for the treatment of patients with iNPH. Notably, LPS surgery is less invasive, and is increasingly being performed in patients with iNPH. This study provides useful and substantial information regarding improvement in CSF-ISF dynamics following LPS surgery. Moreover, this study supports the use of the ALPS method as a potential neuroimaging marker of the glymphatic system and as a method to evaluate the effect of LPS surgery in iNPH.

In the responder subjects, the mean ALPS index in the post-operative group was significantly higher than that in the pre-operative group. On the other hand, in non-responder subjects, the mean ALPS index in the post-operative group was not significantly different from that in the pre-operative group. The brain parenchyma is stretched and compressed by mechanical pressure from ventricular enlargement in patients with iNPH, resulting in neural fibers stretching and normal brain tissue compression, such as blood vessels. Since LPS surgery releases normal tissue obstruction, perfusion and pulsatility may be improved and affect the ALPS index. Besides, responders improved the gait disturbance after LPS surgery and could increase their physical activity; thereby, cerebral pulsatility and blood perfusion might have changed. Mohammadi et al. (48) reported that cerebral pulsatility was reduced after walking. Then, the change of cerebral pulsatility and blood perfusion could influence the measurement of ALPS index and glymphatic clearance (26, 34).

In this study, two participants had decreased mean ALPS index after LPS surgery. One participant was a responder, and another was a non-responder. MMSE scores in both participants were also decreased after LPS surgery. These iNPH participants could have had Alzheimer's pathology. For that reason, both fluid transport and cognitive impairments did not improve even after LPS surgery. Interstitial amyloid-β accumulation is a pathological feature that correlates with poor shunt responsiveness in patients with iNPH (10). Thus, further study on the relationship between ALPS index and amyloid-β accumulation degree, such as in amyloid PET examination, is expected. Additionally, the mean ALPS index was not significantly different between responders and non-responders after LPS surgery. It could be difficult to expect whether the subject is a responder or a non-responder only based on mean ALPS indices in the pre-operative and post-operative states.

In this study, mean ALPS index change was significantly correlated with Evans' index in pre-operative iNPH patients, particularly, in responders. This finding indicates that water diffusivity along perivascular space change of the brain parenchyma with iNPH, especially in responders, recovered in cases of larger ventricular enlargement (6). The brain parenchyma is stretched and compressed by mechanical pressure from ventricular enlargement in patients with iNPH, resulting in normal brain tissue compression, such as blood vessels. A larger ventricular enlargement may increase normal tissue compression in the brain. LPS surgery releases normal tissue compression, and an improvement of mean ALPS index accompanied by normal tissue recovery may be more pronounced in cases with larger ventricular enlargement.

This study has several limitations. First, only a small number of participants were included in this study. Therefore, it is necessary to increase the number of participants to evaluate the glymphatic function of patients with iNPH. Second, 5-mm slice thickness for DWI acquisition in ALPS index measurement in this study was thicker than in some previous papers (21, 34). In the projection area, nerve fibers run in the inferior-superior direction, and it is considered that slice thickness degree is not affected by the accuracy of placing ROIs onto specific fiber tracts. However, in the association area, nerve fibers run in the anterior-posterior direction; thereby, the ROIs placed on the association fiber may be affected by the partial volume effect of other tracts traveling nearby, such as the corpus callosum and the insular cortex. Finally, the ALPS method is not yet a well-established non-invasive tool to measure glymphatic system in living humans. Hence, further studies are needed to establish the correlation between the ALPS index and the ISF excretion function.

## Conclusion

This study demonstrates the improved water diffusivity along perivascular space in patients with iNPH post-LPS surgery. This could be indicative of glymphatic function recovery following the LPS surgery.

## Data Availability Statement

The original contributions presented in the study are included in the article/supplementary material, further inquiries can be directed to the corresponding author.

## Ethics Statement

The studies involving human participants were reviewed and approved by the Ethics Committee of Juntendo University. The patients/participants provided their written informed consent to participate in this study.

## Author Contributions

JK, KKam, TT, and CAn conceived and designed the analysis, analyzed and interpreted the data, drafted, and revised the manuscript for intellectual content. KT, WU, YS, and MN performed data acquisition, analyzed and interpreted the data, and revised the manuscript for intellectual content. All authors read and approved the final manuscript.

## Funding

This work was supported in part by the Japan Society for the Promotion of Science (JSPS) KAKENHI grant numbers 20K16737, 20K09398, 18H02772, 18H02916 and the Juntendo research branding project.

## Conflict of Interest

The authors declare that the research was conducted in the absence of any commercial or financial relationships that could be construed as a potential conflict of interest.

## Publisher's Note

All claims expressed in this article are solely those of the authors and do not necessarily represent those of their affiliated organizations, or those of the publisher, the editors and the reviewers. Any product that may be evaluated in this article, or claim that may be made by its manufacturer, is not guaranteed or endorsed by the publisher.
